# The Application of Rat Models in *Staphylococcus aureus* Infections

**DOI:** 10.3390/pathogens13060434

**Published:** 2024-05-21

**Authors:** Hongyue Liang, Yadong Wang, Fang Liu, Guangcai Duan, Jinzhao Long, Yuefei Jin, Shuaiyin Chen, Haiyan Yang

**Affiliations:** 1 Department of Epidemiology, School of Public Health, Zhengzhou University, Zhengzhou 450001, China; lhy0204@gs.zzu.edu.cn (H.L.); fliu@zzu.edu.cn (F.L.); gcduan@zzu.edu.cn (G.D.); ljz069@zzu.edu.cn (J.L.); jyf201907@zzu.edu.cn (Y.J.); sychen@zzu.edu.cn (S.C.); 2 Department of Toxicology, Henan Center for Disease Control and Prevention, Zhengzhou 450016, China; wangyd76@163.com

**Keywords:** *Staphylococcus aureus*, rat, animal model, infection

## Abstract

*Staphylococcus aureus* (*S. aureus*) is a major human pathogen and can cause a wide range of diseases, including pneumonia, osteomyelitis, skin and soft tissue infections (SSTIs), endocarditis, mastitis, bacteremia, and so forth. Rats have been widely used in the field of infectious diseases due to their unique advantages, and the models of *S. aureus* infections have played a pivotal role in elucidating their pathogenic mechanisms and the effectiveness of therapeutic agents. This review outlined the current application of rat models in *S. aureus* infections and future prospects for rat models in infectious diseases caused by *S. aureus*.

## 1. Introduction

*Staphylococcus aureus* (*S. aureus*) is one of the most widespread and notorious bacterial pathogens as well as a major human pathogen responsible for significant morbidity and mortality worldwide, it can cause a wide range of diseases, including skin and soft tissue infections (SSTIs), pneumonia, endocarditis, osteomyelitis, bacteremia, and sepsis [1,2,3,4]. Approximately 20% of the human population are stably colonized while 30% are colonized in a variable manner [5]. According to a study in the United States, nearly 120,000 *S. aureus* bloodstream infections and 20,000 associated deaths occurred in 2017 [6]. A major concern in the clinical management of *S. aureus* is the bacterium’s powerful ability to develop resistance to antibiotic treatment [7,8]. Since the 1960′s, methicillin-resistant *S. aureus* (MRSA), a main cause to bacterial infections in health care and the community environment, emerged and spread throughout the world, as well as complicating the treatment of *S. aureus* infections and creating a significant economic burden [9,10]. The Institute of Medicine (IOM) of the National Academy of Sciences listed comparative effectiveness research for MRSA as one of its top 25 priorities for national investment, and *S. aureus* was the only bacterial pathogen mentioned in the IOM report [5]. *S. aureus* has the ability to resist the available arsenal of antibiotics and the spread of drug-resistant *S. aureus* interferes with routine treatment options for *S. aureus* infections [11,12]. To date, a host of vaccines against* S. aureus* have yet to show efficacy in humans [13,14,15]. Therefore, there is an urgent need to develop new and efficacious preventive and therapeutic approaches to curb the prevalence of *S. aureus*.

**Figure 1:**
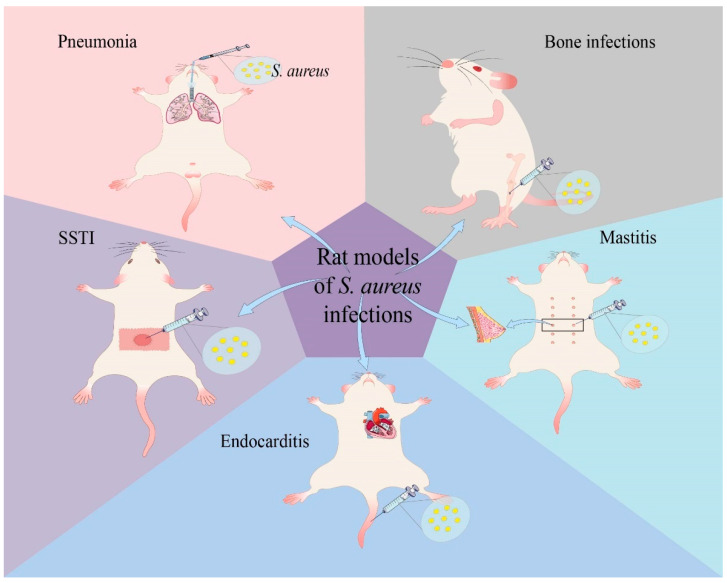
Rat models of *S. aureus* infections. There are several rat models of diseases induced by *S. aureus*, including pneumonia, bone infections, SSTI, endocarditis, and mastitis.

In almost all eras of human history, the use of animals for scientific purposes has been valued. The earliest experiences described by some authors may date back to the 4th century BC in ancient Greece. Since then, and well into the 19th century, animal experiments continued to be carried out [16,17,18]. Around the 20th century, mice and rats became more and more widely used as animal models, and gradually surpassed other animals, including cats, dogs, pigs, and ferrets [19]. Animal models of *S. aureus* infections have played a crucial role in elucidating its pathogenic mechanism and testing the effectiveness of vaccines and therapeutic drugs. Some researchers have chosen rats because they are easier to manipulate compared to larger animals, and they are cheap, in plentiful supply, as well as being easily kept for long periods of time during experiments [20,21]. A host of rat models of *S. aureus* infections have been established, including pneumonia [22], SSTIs [23], osteomyelitis [24], endocarditis [25], and mastitis [26]. (Figure 1). Therefore, this review summarizes the use of rat models in *S. aureus* infections. The advantages and disadvantages of using rat models in infectious diseases caused by *S. aureus* and the future prospects of this approach are also discussed.

## 2. Pneumonia

*S. aureus* has a strong ability to adapt to the respiratory environment, which can cause serious respiratory infections [27,28], and MRSA is also one of the most usual reasons of hospital-acquired and ventilator-associated pneumonia [29,30]. There have been a number of studies reporting the application of rat models in pneumonia induced by *S. aureus* infections. The relatively large organ size of the rat makes surgery and dissection of small anatomical structures easier [31].

### 2.1. Virulence Regulators of S. aureus in Pneumonia

*S. aureus* infections are becoming increasingly difficult to treat due to the prevalence of antibiotic resistant strains [32]. Therefore, it is important to better understand the molecular basis of *S. aureus* virulence in pneumonia, which may be useful for investigating novel therapeutic strategies. Previous studies have shown that fibronectin binding proteins can mediate the internalization of *S. aureus* into non-phagocytic cells, and both alveolar epithelial type I and type II cell surfaces have many potential fibronectin-binding receptors [33,34]. To explore whether fibronectin-binding proteins (fnbp*s*) are virulence factors in the pathogenesis of pneumonia, McElroy et al. established a rat model of pneumonia induced by *S. aureus* [35]. After anesthetizing the rat, a catheter was inserted into the distal airway of the rat, and then *S. aureus* (approximately 10^8^ CFU/0.5 mL) was dripped along the catheter into the distal airway. The results showed that fibronectin binding proteins could influence the degree of internalization of *S. aureus* into alveolar epithelial cells, as the degree of internalization of strain DU5883, which did not express *fnbA* and *fnbB* genes, was 20 times less than that of the wild-type strain 8325-4. While DU5883 (pFnBPA4), which overexpressed *fnbA*, was 2.4 times more internalized than 8325-4. However, compared to rats vaccinated with 8325-4 and DU5883 (pFnBPA4), *S. aureus* growth and the degree of lung damage were increased in rats vaccinated with the deletion mutant DU5883, suggesting that fibronectin-binding protein-mediated internalization of alveolar epithelial cells was not a virulence mechanism in a rat model of pneumonia. Instead, it can reduce the virulence of *S. aureus* in pneumonia, consistent with the results of Flock and Darouiche [36,37].

### 2.2. Preclinical Experiments of Antibacterial Strategies of S. aureus Pneumonia

To explore the efficacy of iclaprim in treating lung infections induced by MRSA, David et al. established a model of pulmonary infection in rats with neutrophil deficiency induced by MRSA embedded in alginate microspheres [38]. Sprague Dawley (SD) rats were preconditioned with cyclophosphamide monohydrate to reduce neutropenic granulocytes firstly, then the rats were anesthetized with isoflurane, and the suspension of alginate bacteria was delivered through intratracheal inoculation (IT) using a stainless-steel feeding needle to infect the rats. Iclaprim significantly reduced the colony counts of MRSA compared with the control group, and the survival rate of rats treated with iclaprim was 100%. In this study, by encapsulating MRSA in alginate, combined with the subsequent biofilm formation, a bacterial growth environment was established which was hard to eradicate and treat, providing a salutary model for testing the effect of antibiotics to treat bacterial infections of the lungs. Josef et al. investigated the therapeutic effect of aerosolized phages on MRSA-induced pneumonia using a rat model [39]. Wistar rats were intubated and ventilated for 4 h. The pneumonia model was established by inoculating the rats with ~ 1 × 10^10^ colony-forming units (CFUs) of MRSA clinical isolate AW7 through the endotracheal tube. It was shown that treatment with aerophages significantly enhanced the survival rate of the rats compared with the control group. In addition, treatment with aerophages combined with intravenous phages rescued 91% of rats compared to either therapy alone. However, the combination of linezolid and aerophages did not show synergistic effects in this study. The findings were consistent with the results of Luca et al., which showed that the combination of intravenous daptomycin and aerophages was not superior to aerophage treatment alone [40].

Different from the methods of constructing rat pneumonia models mentioned above, two studies chose to construct rat pneumonia models by intranasal infection of rats by *S. aureus* [22,41]. Duan et al. used the rat model for the first time to study the protective effects of diphenyl pyrimidine on lung injury induced by *S. aureus* infections. The results showed that treatment with diphenyl pyrimidine could prevent increased mortality induced by *S. aureus* effectively and prevent lung injury by inhibiting the expression of NLRP3 and inflammatory factors in rats [22]. Wu et al. evaluated the influence of isoxanthanol treatment on chronic obstructive pulmonary disease caused by *S. aureus* in a rat model. It was found that isoxanthanol could mitigate chronic obstructive pulmonary disease induced by *S. aureus* in the rat model through suppressing the production of inflammatory cytokines and upregulating the expression of miR-145-5p [41].

Table 1 summarizes the models of *S. aureus*-induced pneumonia in rats, which presents the strains of *S. aureus*, the varieties of rats, the inoculation methods and dose, the substance under study and main results. *S. aureus* 8325-4 and MRSA-AW7 were frequently used to induce pneumonia in rat models. The most commonly used rat breeds were SD and Wistar rats. The modes of infections included endotracheal instillation, endotracheal intubation, and nasal instillation. The inoculation dose of *S. aureus* ranged from 2.6 × 10^5^ CFU to ∼1 × 10^10^ CFU. These studies might provide useful information for the application of rat models in *S. aureus*-induced pulmonary infections in the future.

This table lists the details of the construction of rat models of pneumonia induced by *S. aureus*, and the table contains the surname of the first author, the source of the reference, the *S. aureus* strain, the species of rat used, the method of *S. aureus* injection, the titer of *S. aureus*, the substance under study, and the main results.

## 3. Osteomyelitis/Bone Infections

*S. aureus*-induced osteomyelitis is a complicated disease, which is difficult to treat, as well as a significant complication for orthopaedic patients [21,44]. A variety of animal models have been developed to provide insights into the pathogenesis and treatment of osteomyelitis, including mice, canines, rabbits, and rats [45,46,47]. Rats have distinctive advantages as an animal model of osteomyelitis, their bones are large enough to drill and immobilize, and medullary tubes are of sufficient size for implantation of foreign bodies [21].

### 3.1. Virulence Regulators of S. aureus in Osteomyelitis/Bone Infections

Previous studies have suggested a potential link between Yyc FG (a two-component regulatory system, TCS) and the virulence of MRSA strains in vitro [48]. However, the effect of the Yyc FG pathway on MRSA in vivo is still unclear. In order to further study the function of Yyc FG, Wu et al. established a rat osteomyelitis model, and constructed a clinical strain of MRSA overexpressing AS*yyc*G (AS*yyc*G mutant strain) by using antisense RNA method to investigate the regulatory role of Yyc FG in bacterial biofilm formation and the pathogenicity of MRSA strains [49]. After the rats were anesthetized, their hind legs were shaved and a longitudinal incision of 1 cm in length was made to expose the anteromedial cortex of the rat tibia. Holes with a diameter of 0.1 cm were made with a high-speed grinding drill to expose the medullary cavity. One group was injected with an MRSA bacterial suspension and the other with an AS*yyc*G bacterial suspension. The rats were executed 4 weeks after surgery and bone specimens were taken for further analysis. At 4 weeks, the mRNA expression levels of iNOS, TNF-α, COX-2, and IL-6 were significantly higher in the MRSA group than in the AS*yyc*G group; in particular, COX-2 and IL-6 were twice as high in the MRSA group as in the AS*yyc*G group. This result indicated that the inflammatory response was reduced in the AS*yyc*G group compared with the MRSA group. Aggregated microorganisms were observed in the MRSA group close to the border of the periosteal bone surface and the site of infection, whereas fewer microorganisms were present in the samples of the AS*yyc*G group. The cortex of the rats in the MRSA group was significantly disrupted, accompanied by the infiltration of a large number of inflammatory cells. Gram staining showed that the number of microcolonies in the bone was greater in the MRSA group than in the AS*yyc*G group. The above results suggest that AS*yyc*G might be defective in terms of infection or bone tissue growth, which provides a potential target for the treatment of osteomyelitis induced by MRSA.

### 3.2. Preclinical Experiments of Antibacterial Strategies of S. aureus Osteomyelitis/Bone Infections

In order to more realistically simulate the clinical setting of surgery-associated bone infections, Harrasser et al. established a novel rat model of peri-implant infections of low levels of bacterial inoculation in the tibial metaphyseal [50]. After anesthesia, rats were shaved on both hind limbs, and a skin incision (1 to 2 cm in length) was made at the lateral metaphyseal proximal to the tibia. A single cortical hole of ~8 mm depth was drilled with a 1.8 mm diameter drill, 10 μL of *S. aureus* ATCC 25923 (Group I-IIA: 10^2^ CFU/10 μL, Group I-IIB: 10^3^ CFU/10 μL) was injected into the hole with a microsyringe, and then the implant was immediately inserted into the cavity using a dedicated instrument. Histological sections of the infected tibia of rats showed signs of chronic bone infections, while the control group did not. Meanwhile, when bone fusion was evaluated in rats, extensive osseointegration was found on the surface of hydroxyapatite (HA) and HA–silver (HA-Ag) implants. However, the scores for osseointegration in the infected group were lower compared with the control group, indicating that the presence of *S. aureus* infections reduced bone integration. This rat model can be used for the study of implant-associated bone infections. Besides, Barnea et al. developed a rat model of sternal osteomyelitis induced by *S. aureus* after median sternotomy (MS) to appraise the efficacy of antimicrobial therapies [51]. Rats were firstly subjected to MS incision followed by immediate injection of 1 × 10^7^ CFU/sternum *S. aureus* 18,454 to induce infections. Histopathological examination of the sternum of infected rats on day 10 showed acute osteomyelitis with colonies and soft tissue abscesses compared to the uninfected control group. Since administration of vancomycin significantly reduced *S. aureus* counts compared with the placebo-treated rats, it is also effective against histopathologically confirmed osteomyelitis processes.

In addition, many researchers have used rat models to explore the therapeutic effect of antibiotics on MRSA-induced osteomyelitis [52,53,54]. For instance, Karau et al. compared the efficacy of rifabutin, rifapentine, and rifampin, alone or in combination with vancomycin, in the treatment of foreign body osteomyelitis in rats [52]. The findings indicated that the activity of rifapentine or rifabutin with vancomycin against MRSA were as the same as that of rifampin with vancomycin in a rat model of foreign body osteomyelitis. This suggests that rifapentine and/or rifabutin might be an alternative to rifampin in the clinical management of *S. aureus*-caused periprosthetic joint infections. Karau et al. reported that omadacycline exhibited activity in a rat model of chronic osteomyelitis induced by MRSA when it was administered alone, and higher activity when administered with rifampin [53]. Zhou et al. found that the combination of erythromycin and curcumin was able to inhibit the growth of MRSA and reduce bone infections in rats [54]. It was suggested that the combination of Lysin CF-296/exebacase and daptomycin might provide a novel option for the treatment of acute osteomyelitis on the basis of data from rat osteomyelitis models [44,55].

The rat models of osteomyelitis/bone infections induced by *S. aureus* are summed up in Table 2. Drilling a hole in the tibia with or without implantation of a foreign body followed by injection of *S. aureus* was the most commonly used method to construct a rat model of osteomyelitis/bone infections. However, the titer of *S. aureus* used in these studies was inconsistent. Therefore, more studies are needed to determine which titer is most suitable for establishing the rat model of osteomyelitis/bone infections.

This table lists the details of the construction of rat osteomyelitis/bone infections models induced by *S. aureus*. The table includes the surname of the first author, the source of the reference, the *S. aureus* strain, the species of rat used, the method of model construction, the method of injection of *S. aureus*, and the method of injection of *S. aureus* as well as the titer of *S. aureus*, the substance under study and main results.

## 4. Skin and Soft Tissue Infections (SSTIs)

Skin is one of the usual sites of host immune response to *S. aureus* infections [58]. Rats are larger and therefore have more tissue for physiological and pathological analysis [31]. Their larger back area also makes it easier to maneuver on the skin.

### 4.1. Virulence Regulators of S. aureus in SSTI

*S. aureus* is capable of secreting virulence factors associated with the cell surface, allowing it to infect almost all human tissues [59]. Sae, one of the TCSs of *S. aureus*, is able to regulate the expression of α-toxin by binding to a consensus SaeR binding site upstream of the *hla* promoter [60,61]. Gudeta et al. mutated the SaeR binding sequence upstream of the *hla* promoter (*sbm* mutant) to evaluate its effect on the pathogenesis of *S. aureus* USA300 JE2 using a rat SSTI model. After SD rats were anesthetized, 10^7^ CFU of *S. aureus* in 0.1 mL DBPS were inoculated subcutaneously on both sides of the shaved flanks of the rats to establish the SSTI model [62]. To assess the effect of the deletion of Sae R binding to the *hla* promoter region on Hla production, the levels of Hla production in the wild type and the *sbm* mutant were compared by Western blotting using the *hla::bursa* mutant and *saeR::bursa* mutant (transduced from the corresponding transposon mutant by using phage 52A) as controls. The results showed that the production of α-toxin was significantly reduced in the *sbm* mutant compared with the wild type. The *saeR::bursa* mutant produces similar levels of α-toxin as the *sbm* mutant, while the levels of the *hla::bursa* mutant are undetectable. The sizes of the rats’ abscesses were measured daily. No significant skin necrosis was observed during the 12-day infection period. During this period, the abscesses of the rats in the *sbm* mutant strain group were significantly smaller than those in the wild-type infected group from day 1 to day 7, and the abscesses in the *saeR::bursa* mutants were significantly smaller than in the wild type on days 1 through 8. The rats were sacrificed on day 12 post-infection, and the bacterial load of the abscesses was determined. The bacterial counts in the rats in the *sbm* mutant group were not significantly lower than those in the wild-type group, but were significantly higher than those in rats in the *saeR::bursa* mutant group. The above results suggest that the regulation of *hla* by SaeR played an important role in the pathogenicity of *S. aureus*. However, *S. aureus* virulence factors are complexly regulated and the regulatory factors interact with each other [62]. Therefore, it is difficult to prove the role of specific regulatory pathways in the pathogenic process of *S. aureus*.

### 4.2. Preclinical Experiments of Antibacterial Strategies of S. aureus SSTIs

Atopic dermatitis (AD) is an increasingly common chronic recurrent inflammatory disease [63], and the skin of up to 100% of patients with AD is colonized with *S. aureus* [64]. Moreover, it is reported that the density of *S. aureus* in the skin is correlated with the severity of the disease in AD patients [65]. Han et al. researched the effect of glyoxal exposure using a rat model of AD [66], demonstrating that glyoxal can aggravate pruritus and dermatitis symptoms in AD rats. In addition, exposure to glyoxal elevated the growth of *S. aureus* in the skin of AD rats and induced the colonization of *S. aureus* in that of naive rats.

*S. aureus* is one of the most prevalent strains present in patients with wound infections [67], and antibiotics are widely used to treat wounds [68]. Simonetti et al. used a rat model of MRSA infections to test the effect of daptomycin on burn wound healing compared to that of teicoplanin [69]. The rats were anesthetized with ketamine–xylazine, and their backs were shaved and cleaned. A copper rod heated in boiling water was placed in the paravertebral site of the rat without pressure for 40s. Only the weight of the copper block was used to create the burn. A small piece of gauze was placed at each burn, and the infection model was established by inoculating the wound with 5 × 10^7^ CFU of *S. aureus* ATCC 43300. The results indicated that daptomycin had stronger antibacterial activity compared with teicoplanin, and the rats treated with daptomycin showed better epithelization than those treated with teicoplanin.

Rat skin wound models infected with *S. aureus* have been used to evaluate the effects of plant extracts on wound healing. Rajoo et al. prepared an ointment from the leaf extract of *Elaeis guineensis* and evaluated its wound healing activity in a rat model of *S. aureus* infections [68]. A 1.5 × 1.5 cm full-thickness wound was created on the shaved dorsal area of SD rats and inoculated with 0.1 mL of ~10^9^ CFU of *S. aureus*. This suggested that the methanol extract from the leaf of *E. guineensis* had good antibacterial activity against *S. aureus* in the wound site of rats. Ekom et al. found that methanol extract from the seeds of *Persea americana* showed antibacterial and wound healing activity against *S. aureus* in a rat model [70].

Taulescu et al. investigated the potential of a novel phosphate-based soluble glass component, which contains Zn and different amounts of Cu, Ag, and K, to promote skin wound healing in a rat model [71]. The back and thoracoabdominal regions of the rats were shaved, and 2 symmetrical, full-length excisional wounds 8 mm in diameter were made through the muscle tissue on the back of each rat using a biopsy punch. The results showed that, compared with the control group, the group treated with the phosphate-based soluble glass component had significantly higher regenerative effects in collagen synthesis, angiogenesis, and re-epithelialization according to the in vivo full-thickness wound healing evaluation.

The skin and soft tissue infection models in rats induced by *S. aureus* are shown together in Table 3. The model construction methods used in each study are various, and different methods can be used to induce the rat skin models in future studies.

This table lists the details of the construction of the model of skin and soft tissue infections induced by *S. aureus* in rats. The table contains the surname of the first author, the source of the reference, the *S. aureus* strain, the species of rat used, the method used to create the skin wound, as well as the titer of *S. aureus*, the substance under study, and the main results.

## 5. Endocarditis

*S. aureus* is currently the foremost prevalent pathogen of infective endocarditis (IE) [73]. IE is characterized by an intricate course and high mortality (in-hospital mortality as high as 40%), mainly involving heart valves, endocardium, and synthetic materials [74,75]. Therefore, it is of great necessity to find efficient strategies to treat IE and improve the prognosis of IE patients. Rat endocarditis is an acknowledged experimental animal model, which is close to human native valve endocarditis. Moreover, it has been used to examine the role of specific *S. aureus* virulence factors and the efficacy of antibiotic treatment regimens [76]. Some studies have used rabbit models to research IE; however, rats are more advantageous as experimental animals than rabbits because they are easier to keep and less expensive [77].

### 5.1. Preclinical Experiments of Antibacterial Strategies of S. aureus Endocarditis

Information on the rat model of endocarditis induced by *S. aureus* is detailed in Table 4. Most studies chose to construct a rat endocarditis model by placing a catheter in the left ventricle through the carotid artery of the rat and leaving it in place for 24 h, followed by inoculation through the tail vein with *S. aureus*, which varied in titer from 10^4^ CFU to 4.6 × 10^8^ CFU. For example, Singh et al. used a rat IE model to evaluate the in vivo efficacy of ceftaroline (CPT) against MSSA strains with cefazolin (CFZ) inoculation effect (InE) [78]. It was suggested that CPT was effective against the MSSA strains, and antibacterial regimens with CPT or containing CPT could be effective in patients for whom CFZ treatment had failed. Miller et al. tested the capability of a β-lactamase inhibitor, clavulanic acid, to recover the efficacy of cefazolin against *S. aureus* in a rat model of infective endocarditis. The findings demonstrated that the addition of clavulanic acid to CFZ could eliminate the InE of *S. aureus* TX0117 in a rat model of endocarditis [79]. Lerche et al. used an experimental rat model to evaluate the efficacy of tobramycin as a monotherapy in the treatment of *S. aureus* infective endocarditis. The results suggested that tobramycin could only temporarily decrease the bacterial load and inflammation in the myocardium, valvular vegetation, and parts of the spleen and was insufficient in most cases of rat infective endocarditis infected with *S. aureus* [80]. Subsequently, they further explored the effect of hyperbaric oxygen therapy (HBOT) as an adjuvant therapy to the efficacy of tobramycin [75], which showed that HBOT significantly decreased the bacterial load in the valves, myocardium and spleen, and the weight of vegetation by increasing the effectiveness of tobramycin in comparison with the non-HBOT group. Furthermore, host inflammatory response was decreased, and clinical status was improved significantly compared to the non-HBOT group.

This table lists the details of the construction of rat endocarditis models induced by *S. aureus*. The table includes the surname of the first author, the source of the reference, the *S. aureus* strain, the species of rat used, the location of the catheter, the mode of inoculation, the titer of *S. aureus*, the substance under study and main results.

There is a study in which, in order to determine the optimal inoculum for inducing IE, rats were inoculated with 10^4^, 10^5^, or 10^6^ CFU of *S. aureus* through the tail vein 7 days after catheter insertion. Xiong et al. applied bioluminescence in an in vivo imaging system (IVIS) to IE, using a rat model to track the infection in living animals and evaluate the real-time effectiveness of some common anti-staphylococcal drugs [82]. In the case of the vancomycin treatment group, cardiac bioluminescent signals (BLS) decreased on the 1st day after treatment, and during treatment, BLS continued to decline. However, compared with the images at the end of treatment, there was a gradual increase in BLS during the 2 days after vancomycin treatment was discontinued, suggesting microbiological relapse. The analysis of regression revealed a significant correlation between average heart BLS intensity and *S. aureus* density in vegetation in antibiotic-treated and untreated control animals. In animal experimental models, common methods of monitoring pathogens are complicated and often require tissue removal or animal sacrifice. Therefore, IVIS may be able to overcome these limitations and provide substantial progress in the treatment of *S. aureus* infections in the future.

### 5.2. Virulence Regulators of S. aureus in Endocarditis

*S. aureus’*s success as a pathogen is partly due to its ability to express a large number of virulence factors [3]. Most bacterial factors associated with the degree of infection are mobile genetic elements (MGEs) belonging to accessory genomes, include temperate phages that are integrated stably within the bacterial genome, and studies have shown that many *S. aureus* clinical isolates have one to several prophages in their genomes [84]. Laumay et al. used an experimental model of endocarditis in rats to evaluate the contribution of prophage to the virulence of *S. aureus* [84]. Aortic vegetations were generated using a catheter inserted through the aortic valve via the right carotid artery of the female Wistar rat, and the rats were then intravenously injected with 10^4^ CFU *S. aureus* for exponential phase culture. Rats were sacrificed 24 h after inoculation and vegetation quantitative cultures were performed on blood agar plates. Strains S123 and S124 are ancestral isolates that colonized livestock asymptomatically and lacked prophages, and strain S1 contains two prophages. The phage was introduced from S1 into S123 to obtain strain S123Sa2, and similarly to obtain strain S124Sa2. Compared with parental strains S123 and S124, lysogenic strains S123Sa2 and S124Sa2 showed increased adherence to human fibrinogen by 12- and 1.5-fold, respectively. Compared with S123, the expression of *clfA*, the gene responsible for adhesion fibrinogen, was higher in S123Sa2. There was also a moderate increase in *fnbpA* and *cna* in S124Sa2 compared with S124. S123 and S124 exhibited limited internalization (8-fold and 2.5-fold lower, respectively) compared with S123Sa2 and S124Sa2. In addition, compared to the parental strain S123, the infection rate of rats was significantly increased after inoculation with the S123Sa2 strain. S123 obtained the lowest number of bacteria in vegetation, while all other strains containing prophage showed higher titers of bacterial vegetation. This study shows that prophages have a direct effect on the virulence and pathogenicity of *S. aureus*.

## 6. Mastitis

Mastitis is an inflammatory response caused by breast tissue infections, which can be caused by a variety of bacteria [85]. And *S. aureus* is one of the most common pathogens of bovine infectious mastitis, as well as intramammary infections of dairy goats, which is widespread in many countries, seriously restricting the development of the global dairy industry [86,87,88]. Prevention and control of mastitis has been a significant challenge for the dairy industry [89]. As the standard experimental animal, the rat model was chosen to study mastitis because its papillary ducts and mammary glands are relatively large and can be inoculated without the aid of a microscope [90].

In order to explore the mechanism of mastitis caused by *S. aureus* and to evaluate the preventive and therapeutic effects of novel strategies, rat models of *S. aureus* mastitis have been reported. Table 5 lists the information on the reported models of *S. aureus* mastitis in rats. The model of mastitis is usually established by using parturient rats, and the postpartum time varies from 3 to 8 days. The titer of *S. aureus* inoculated ranged from 2 × 10^2^ CFU to 2 × 10^7^ CFU. The range of infection duration varied from 6 h to 96 h. But equally, these studies were performed by injecting *S. aureus* into the fourth pair of mammary tissues through mammary ducts. Significant inflammatory changes could be observed in the mammary tissue of the infected rats, with neutrophils and macrophages infiltrating the mammary alveoli, ducts, perivascular, and connective tissues.

This table lists the details of the construction of the model of mastitis induced by *S. aureus* in rats. The table contains the surname of the first author, the source of the reference, the *S. aureus* strain, the species of rat used, the postpartum days of the rat, the location of the rat’s mammary gland inoculated with *S. aureus*, the titer of *S. aureus*, the substance under study, and the main results.

### 6.1. Pathogenic Mechanisms of S. aureus in Mastitis

Several studies have investigated the potential mechanisms of mastitis caused by *S. aureus* in rat models. The study by Cai et al. explored the proteomic changes in *S. aureus*-infected rat breast tissue [91]. Data from the Kyoto Encyclopedia of Genes and Genomes Enrichment and Gene Ontology analyses showed that there was an association between upregulated differentially significantly expressed proteins including alpha-2-macroglobulin, inter-α-trypsin inhibitor-heavy chain 4, and integrin alpha M with immune responses. This suggested that the rat model could be used to better understand the pathogenesis of mastitis caused by *S. aureus* infections. Wang et al.’s study suggested that TLR2 was an important immune recognition receptor activated in rat breast tissue infected with *S. aureus* and that high NOD2 expression in breast tissues was a marker of *S. aureus* recognition and immune response [92]. These results can help us to understand the molecular mechanism of *S. aureus-*induced mastitis.

### 6.2. Preclinical Experiments of Antibacterial Strategies of S. aureus Mastitis

Furthermore, some researchers have used rat models to study the effects of novel strategies on improving and protecting against *S. aureus*-induced mastitis. Tong et al. prepared a novel berberine hydrochloride carboxymethyl chitosan hydrogel (BH-CMCH) to evaluate its potential for the prevention and treatment of mastitis induced by *S. aureus* in a rat model [26]. Zhu et al.’s study suggested that CpG-DNA, a kind of non-methylated and specific DNA motif including cytosine-phospho-guanine dinucleotide, could induce the production as well as accelerate the release of IL-6, which promotes responses to *S. aureus* infections in rats [90]. Besides, CpG-DNA induced a rapid infiltration of more polymorphonuclear leukocytes, ensuring therapid clearance of *S. aureus* from breast tissue and shortening the time to inflammation development. Liu et al. used a rat mastitis model to study the protective effect of organic selenium on *S. aureus*-induced mastitis [93]. It was found that organic selenium might regulate the inflammatory response by suppressing the activation of the NF-κB signaling pathway and attenuating the inflammatory reaction to infections by modulating the MAPK signaling pathway. Zhang et al.’s study found that the anti-inflammatory effect of salvia miltiorrhizae polysaccharides (SMPs) on mastitis induced by *S. aureus* in rats might be related to the inhibition of the activation of NF-κB and MAPK signaling pathways and thus the inhibition of the expression of inflammatory cytokine genes [85].

However, it has been found that there are obvious differences in the immune reactivity of the mammary glands of rats and cows during mastitis caused by *S. aureus* [85]. Therefore, future studies should be further conducted in dairy cows.

## 7. Others

In addition to the above-mentioned models, researchers have utilized rat models to investigate various other diseases induced by *S. aureus*, including bacteremia [94], sepsis [95], endometritis [96], and keratitis [97].

*S. aureus* is the leading cause of bacteremia and can cause noteworthy morbidity and mortality [98,99]. Channabasappa et al. investigated the efficacy of P128, a bacteriophage-derived chimeric ectolysin, in a rat model of bacteremia caused by *S. aureus* [94]. Female Wistar albino rats were intravenously injected with 10^9^ CFU of MRSA USA300. The CFU counts of MRSA USA300 in blood were found to be between 2 × 10^8^ ± 8 × 10^7^ CFU/mL in the first minute following MRSA USA300 injection. The numbers dropped by one to three orders of magnitude by 4 h. The CFU counts in the blood stayed in the range from 2 × 10^4^ CFU/mL to 2 × 10^6^ CFU/mL for the next four days. At 96 h postinfection, the CFU counts were 1.89 × 10^7^ ± 9.84 × 10^6^ in the kidney, 2.37 × 10^5^ ± 2.19 × 10^5^ in the liver, 2.16 × 10^4^ ± 1.26 × 10^4^ in the spleen, and 2.02 × 10^4^ ± 1.93 × 10^4^ in the lung, respectively. In the kidneys, diffuse abscesses were seen after gross necropsy. Treatment with P128 contributed to a dose-dependent improvement in survival, and P128 treatment (2.5 mg/kg) caused little, or no abscesses compared with rats treated with saline. The mean body weight of rats treated with P128 (2.5 mg/kg) increased significantly on day 7 compared with the control group. Accordingly, P128 can be used as a new option for the treatment of *S. aureus* bacteremia. This rat model of bacteremia is reproducible and exhibits gradual disease progression, which can lead to death over several days, and therefore more closely resembling human infections.

Sepsis is a systemic inflammatory reaction with high mortality [100]. The load of pathogens in the blood correlates with the severity and mortality of disease in patients with sepsis [101]. Kang et al. developed an external blood purification device, based on the biospleen microstructure of rats, capable of removing pathogens such as *S. aureus* from the flowing blood of sepsis patients [95]. Rats were injected intraperitoneally with 1 mL of PBS containing *S. aureus* (ATCC 12598, 5 × 10^8^ CFU/mL). Pathogen levels in the blood of rats increased 3-4 h after injection and peaked at about 10 h. At 10 h, the rats were anesthetized, the jugular vein catheter of the rats was connected to the tubing of the biospleen device, and saline containing heparin, magnetic opsonin, and magnetic beads was injected using a syringe pump at a flow rate of 7.1 μL min^−1^. The findings indicated that the biospleen could clear >90% of *S. aureus* from the blood, decrease the load of *S. aureus* and immune cell infiltration in multiple organs, and reduce the levels of several inflammatory cytokines (such as interleukin-1α, interleukin-4, interleukin-6, interferon-γ, and granulocyte-macrophage colony-stimulating factor) in a rat sepsis model caused by *S. aureus*.

*S. aureus* infection of the endometrium usually causes serious uterine disease in humans and animals, endometritis is a reproductive disorder that widely exists in female domestic animals [102,103]. Jia et al. used a rat model to investigate the function of nisin in ameliorating endometritis induced by *S. aureus* [96]. Female SD rats were anesthetized on postnatal day 4 and inoculated transvaginally with 1 × 10^8^ CFU of *S. aureus* to induce endometritis. Two days after that, the rats were administrated with nisin (25 mg/kg), kanamycin (30 mg/kg), or water (control group) for 7 days, respectively. Compared with the control group, the uterine weights of the rats in the nisin and kanamycin groups were relatively lower, indicating that nisin and kanamycin could inhibit the proliferation of *S. aureus* in uterine smooth muscle. In addition, they also promoted the restoration of endometrial structure and normalization of neutrophils in rats. Furthermore, the ratios of TNF-α/IL-2 and IL-6/B_7-2_ were also significantly decreased in the nisin group, suggesting that nisin may be a possible immunomodulator.

*S. aureus* is one of the main causative agents of keratitis [104]. To explore the antibacterial efficacy and ocular safety of URP20, an antimicrobial peptide, Li et al. established a rat model of *S. aureus*-induced keratitis [97]. Before the start of the experiment, the eyes of SD rats were examined by slit lamp to ensure that there were no defects. Rats were randomly divided into a control group (A), an antibiotic treatment group (B), and an URP20 treatment group (C). Afterwards, the rats were anesthetized and the corneal epithelium of the left cornea of the rats was scraped with a 26-gauge needle to create a superficial wound, but without disrupting the stromal layer. A suspension of approximately 10^7^ CFU of *S. aureus* was immediately inoculated on the corneal surface to infect the rats. After infection, rats in group A were covered with plexiglass sheets to prevent bacterial fluid loss, their upper and lower eyelids were sutured, the sutures were removed after 24 h, and saline drops were administered every 2 h, six times a day for 2 consecutive days. Cefazolin eye drops were added to group B, and URP20 eye drops were added to group C. The rest of the operation was the same as that in group A. The number of normal ocular epithelial cells was reduced in group A rats, with a loss of nuclei and heterogeneous cell morphology. The number of normal epithelial cells was increased in group B rats, with a small number of cells invading into the cell interior. The number of normal epithelial cells was increased in group C rats, and there was no obvious bacterial invasion inside the cells, but some of the epithelial cells showed nuclear anisotropy. Opacification and vascular hyperplasia were seen in group A rats 72 h after *S. aureus* infections, and large areas of bacterial invasive damage were visible under cobalt blue-light irradiation. The area of fluorescein sodium staining in group C decreased after URP20 treatment. The above results indicate that URP20 can be used as a candidate drug for the treatment of ocular infections, which is worthy of further investigation.

## 8. Conclusions and Perspective

This review summarized the current application of rat models in *S. aureus* infectious diseases, including pneumonia, osteomyelitis/bone infections, skin and soft tissue infections, endocarditis, mastitis, bacteremia, and so forth. Tremendous progress has been made since the start of research on *S. aureus*, thanks in part to the use of the rat models as rats have multiple superiorities in modeling human diseases: not only are rats amenable to surgical procedures compared to mice, but also their larger blood volume allows continuous blood collection from the same animal [105]. About 90% of rat genes have strict immediate homologs in both the mouse and human genomes [106]. The larger size of the rat and the correspondingly larger vascular, cardiac, blood, and urine volumes allow for more complex physiological measurements and surgical maneuvers [107]. As a promising preclinical model, the rat model has provided many insights in understanding the pathogenic mechanism of *S. aureus* and exploring therapeutic approaches, which also promotes the research of *S. aureus* vaccines. In this review, we enumerate many advantages of the rat model, but of course, rats are not suitable for all types of studies. For example, murine and lung neutropenic infection models are standard models for studying PK/PD target development and comparative pharmacodynamics [108,109,110]. Moreover, the rat also has some disadvantages as an experimental model, housing for rats is sometimes three times more expensive than the cost of housing for mice, and it requires a much larger space for the experimental setup [107]. The weight of the rat is also detrimental to the drug development, as rats need to use more of the compound to achieve the same dose as mice [107]. More importantly, there are essential differences between rats and humans in the immune response to *S. aureus*. Consequently, more human-like animal models should be further searched for research in the future. No single species is ideal in all respects, and weighing the advantages and disadvantages of different animal models is needed [111]. In addition, it has been found that patients with pre-existing comorbidities may have more severe conditions when infected with *S. aureus*. For example, *S. aureus* endocarditis is associated with higher mortality in patients with diabetes than in those without diabetes [112]. However, knowledge about the impact of comorbidities in animal models is very limited. Therefore, rat models suitable for the study of different complications may be constructed in the future by inducing additional disease models or by hybridization with existing models such as diabetic rats and rats with cardiovascular diseases.

## Figures and Tables

**Table 1:** Summary of rat models used for the study of *S. aureus* pneumonia.

Ref.	*S. aureus* Strain	Species of Rat	Inoculation Method	Titer/CFU	The Substance under Study	Main Results
Huang [38]	AH1252	Sprague Dawley (SD)	Endotracheal instillation	2.6 × 10^5^	Iclaprim	Iclaprim significantly reduced the colony counts of MRSA compared with the control group, and the survival rate of the rats treated with iclaprim was 100%.
Prazak [39]	MRSA-AW7	Wistar	Endotracheal intubation	~1 × 10^10^	Aerosolized Phages	Treatment with aerophages enhanced the survival rate of rats compared with the control group significantly, and aerophages combined with intravenous phages rescued 91% of rats compared to either therapy alone.
Valente [40]	MRSA-AW7	Wistar	Endotracheal intubation	~1 × 10^10^	Daptomycin, phage therapy	Intravenous daptomycin combined with aerosolized phage treatment improving survival and reducing bacterial load in the lungs or spleen of rats was not superior to aerophage treatment alone.
Duan [22]	8325-4	SD	Nasal instillation	4 × 10^8^	Diphenyl pyrimidine	Treatment with diphenyl pyrimidine could prevent increased mortality induced by *S. aureus* effectively and prevent lung injury by inhibiting the expression of NLRP3 and inflammatory factors in rats.
Wu [41]	8325-4	-	Nasal instillation	4 × 10^8^	Isoxanthanol	Isoxanthanol could mitigate chronic obstructive pulmonary disease induced by *S. aureus* in the rat model through suppressing the production of inflammatory cytokines and upregulating the expression of miR-145-5p.
Mcelroy [35]	8325-4 DU5883	-	Endotracheal intubation	~1 × 10^8^	Fnbp mutant	Compared with rats vaccinated with 8325-4 and DU5883 (p fnbpA 4), *S. aureus* growth and the degree of lung damage were increased in rats vaccinated with the deletion mutant DU5883, suggesting that fibronectin-binding protein-mediated internalization of alveolar epithelial cells could reduce the virulence of *S. aureus* in pneumonia.
Niu [42]	MRSA B3180	SD	Endotracheal intubation	2.7 × 10^8^	Colistin	All the rats in the infected group developed pneumonia with a marked cellular inflammatory response compared with the negative control group. In contrast to the untreated controls, rats in the colistin and SB203580 + colistin groups showed a reduction in alveolar septal thickening and cellular inflammatory infiltration.
Montgomery [43]	USA400 USA300	SD	Endotracheal intubation	(low inoculum) 4–5 × 10^8^ (high inoculum) 1–2 × 10^9^	Virulence of USA300 and USA400	In a rat necrotizing pneumonia model, USA300 isolates were more virulent than USA400.
Mcelroy [28]	8325-4 DU1090	SD	Intubated through a tracheotomy	8325-4 (6.1 ± 1.1) × 10^8^ (3.53 ± 0.36) × 10^9^ DU1090 (4.04 ± 0.87) × 10^9^	α-toxin	α-toxin is an important cause of air–blood barrier damage in vivo, but it may not act directly on alveolar epithelial type I cells.

**Table 2:** Summary of rat models used for the study of *S. aureus* osteomyelitis/bone infections.

Ref.	*S. aureus* Strain	Species of Rat	Method of Construction	Inoculation Method	Titer/CFU	The Substance under Study	Main Results
Wu [49]	MRSAASyycG	SD	Drill a 0.1 cm diameter hole with a high-speed grinding drill	Intraosseous injection	-	YycFG two-component pathway	AS*yyc*G may be defective in terms of infection or bone tissue growth, which provides a potential target for the treatment of osteomyelitis induced by MRSA.
Harrasser [50]	ATCC 25923	Wistar	Drill the tibia, implant titanium screw	Microsyringe Injection	1 × 10^2^ 1 × 10^3^	A new model of implant-related osteomyelitis	This rat model can be used for the study of implant-associated bone infections.
Barnea [51]	18454	-	Median sternotomy	Intraosseous injection	1 × 10^7^	A new *S. aureus* mediastinitis and sternal osteomyelitis model	Histopathological examination of the sternum of infected rats on day 10 showed acute osteomyelitis with colonies and soft tissue abscesses compared to the uninfected control group.
Karau [52]	MRSA IDRL-6169	Wistar	Drill the tibia, implant Kirschner wire	Intraosseous injection	1 × 10^6^	Rifabutin, rifapentine	Rifapentine and/or rifabutin might be an alternative to rifampin in the clinical management of *S. aureus*-caused periprosthetic joint infections.
Karau [53]	MRSA IDRL-6169	SD	Drill the tibia	Intramedullary injection	6 × 10^6^	Omadacycline	Omadacycline showed activity when administered alone and was even more active when administered with rifampin, eliminating the occurrence of rifampin resistance in rifampin monotherapy.
Zhou [54]	ATCC 43300	Wistar	Drill the tibia, implant Kirschner wire	Intramedullary injection	1 × 10^8^	Erythromycin, curcumin	The combination of erythromycin and curcumin was able to inhibit the growth of MRSA and reduce bone infections in rats.
Karau [44]	MRSA IDRL-6169	SD	Direct injection	Intraosseous injection	1 × 10^7^	Lysin CF-296, Daptomycin	CF-296 is active and well tolerated in rats with osteomyelitis when used with daptomycin.
Karau [55]	MRSA IDRL-6169	SD	Direct injection	Intraosseous injection	1 × 10^7^	Exebacase, daptomycin	Daptomycin plus exebacase is more effective than exebacase or daptomycin alone.
Vergidis [56]	MRSA IDRL-6169	Wistar	Drill the tibia, implant a stainless-steel wire	Intraosseous injection	1 × 10^6^	Vancomycin, tigecycline, rifampin	Treatment with rifampin plus tigecycline resulted in a significant reduction in bacterial counts.
Mills [57]	ATCC 12600, MRSA, MRSE	Wistar	Create a mid-femoral fracture in the right distal femur of the rat, then open the fracture site and dissect the periosteum around the fracture to create an open injury	-	1 × 10^4^	CSA-90	The prophylactic use of CSA-90 can reduce infection and promote bone healing of open fractures.

**Table 3:** Summary of rat models used for the study of *S. aureus* skin and soft tissue infections.

Ref.	*S. aureus* Strain	Species of Rat	Method of Wound Creation	Titer/CFU	The Substance under Study	Main Results
Gudeta [62]	See the original table for details	SD	-	1 × 10^7^	Assess the effect of the deletion of Sae R binding to the *hla* promoter region on *hla* production	The production of α-toxin was significantly reduced in the *sbm* mutant compared with the wild type. The *saeR::bursa* mutant produces similar levels of α-toxin as the *sbm* mutant.
Simonetti [69]	ATCC 43300	Wistar	Burn with a copper rod	5 × 10^7^	Daptomycin	Daptomycin had stronger antibacterial activity compared with teicoplanin, and the rats treated with daptomycin showed better epithelization than those treated with teicoplanin.
Rajoo [68]	-	SD	Full thickness wound (1.5 × 1.5 cm)	~1 × 10^9^	*Elaeis guineensis* Jacq leaves	The methanol extract from the leaf of *E. guineensis* had good antibacterial activity against *S. aureus* in the wound site of rats.
Ekom [70]	ATCC 25923 *S. aureus* 56/18	Wistar	Excision of the skin 2 cm in diameter made by scalpel and sharp scissors	1.5 × 10^7^	Methanol extract from the seeds of *Persea americana*	Methanol extract from the seeds of *P. americana* showed antibacterial and wound healing activity against *S. aureus* in a rat model.
Taulescu [71]	ATCC 25923	Wistar	Two full-thickness excision wounds with a diameter of 8 mm created by a biopsy punch	-	A novel therapeutic phosphate-based glass	Compared with the control group, the group of the phosphate-based soluble glass component had significantly higher regenerative effects in collagen synthesis, angiogenesis, and re-epithelialization according to the in vivo full-thickness wound healing evaluation.
Muller [72]	ATCC 25923 MRSA	Wistar	The wound was induced with a metal perforator with a diameter of 8 mm until the myofascial membrane was exposed	-	*Sebastiania hispida* gel	ExtEtOH gel based on *S. hispida* shoots can be used to treat infected wounds as a complementary therapy for the closure of infected wounds.

**Table 4:** Summary of rat models used for the study of *S. aureus* endocarditis.

Ref.	*S. Aureus* Strain	Species of Rat	Location of Catheter	Mode of Vaccination	Titer/CFU	The Substance under Study	Main Results
Singh [78]	TX0117, TX0117c	SD	Left ventricle	Tail vein injection	4.6 × 10^8^ 2.4 × 10^8^	Ceftaroline (CPT)	CPT was effective against the MSSA strains, and antibacterial regimens with CPT or containing CPT could be effective in patients for whom cefazolin (CFZ) treatment had failed.
Miller [79]	TX0117	SD	Left ventricle	Tail vein injection	-	Clavulanic acid	The addition of clavulanic acid to CFZ could eliminate the InE of *S. aureus* TX0117 in a rat model of endocarditis.
Lerche [80]	NCTC 8325-4	Wistar	-	Tail vein injection	0.5 × 10^7^	Tobramycin	Tobramycin could only temporarily decrease the bacterial load and inflammation in the myocardium, valvular vegetation, and parts of the spleen.
Lerche [75]	NCTC 8325-4	Wistar	Aortic valve	Intravenous inoculation	0.5 × 10^7^	Hyperbaric oxygen therapy (HBOT)	HBOT significantly decreased the bacterial load in the valves, myocardium and spleen, and the weight of vegetations by increasing the effectiveness of tobramycin in comparison with non-HBOT group.
Save [81]	A panel of 63 *S. aureus* strains isolated from humans and animals	Wistar	Superior vena cava	Intravenous inoculation	1.30 ± 0.35 × 10^5^	Bacteriophages, flucloxacillin	Bacteriophages in combination with a subtherapeutic dose of flucloxacillin was highly synergistic against experimental *S. aureus* endocarditis, superior to either phage or flucloxacillin alone.
Xiong [82]	Xen29	SD	Left ventricle	Tail vein injection	10^4^, 10^5^, 10^6^	*In vivo* imaging system (IVIS)	The analysis of regression revealed a significant correlation between average heart bioluminescent signals (BLS) intensity and *S. aureus* density in vegetation in antibiotic-treated and untreated control animals.
Nannini [83]	TX0117, TX0117c	SD	Left ventricle	Tail vein injection	-	Cefazolin (CFZ), daptomycin, nafcillin	In strain TX0117 group, daptomycin and nafcillin were both significantly better than cefazolin in reducing *S. aureus* count, and CFZ significantly reduced vegetation titer in the TX0117c group.
Laumay [84]	*S. aureus* from the CC398 lineage	Wistar	Aortic valve	Intravenous inoculation	1 × 10^4^	Temperate prophages	Compared with parental strains S123 and S124, lysogenic strains S123Sa2 and S124Sa2 showed increased adherence to human fibrinogen.

**Table 5:** Summary of rat models used for the study of *S. aureus* mastitis.

Ref.	*S.aureus* Strain	Species of Rat	Postpartum/Days	Mammary Glands	Titer/CFU	The Substance under Study	Main Results
Cai [91]	ATCC 29740	SD	3	L4 and R4	2 × 10^6^	Proteomic changes	There was an association between upregulated differentially significantly expressed proteins including alpha-2-macroglobulin, inter-α-trypsin inhibitor-heavy chain 4, and integrin alpha M with immune responses.
Wang [92]	YZ 20108	Wistar	4	L4 and R4	2 × 10^6^	TLR2, NOD2, and related cytokines	TLR2 was an important immune recognition receptor activated in rat breast tissue infected with *S. aureus* and that high NOD2 expression in breast tissues was a marker of *S. aureus* recognition and immune response.
Tong [26]	ATCC 29740 ATCC 29213	SD	3	L4 and R4	2 × 10^6^	Berberine hydrochloride carboxymethyl chitosan hydrogel (BH-CMCH)	BM-CMCH may heighten the degradation of autophagolysosomes and decrease the amount of *S. aureus* in mammary epithelial cells by activating lysosomes and upregulating the expression of relevant proteins.
Zhu [90]	CMCC 26081	SD	3	L4 and R4	2 × 10^2^ 2 × 10^4^	CpG-DNA	CpG-DNA induced a rapid infiltration of more polymorphonuclear leukocytes, ensuring rapid clearance of *S. aureus* from breast tissue and shortening the time to inflammation development.
Liu [93]	-	Wistar	4	L4 and R4	2 × 10^6^	Organic selenium	Organic selenium might regulate the inflammatory response by suppressing the activation of the NF-κB signaling pathway and attenuating the inflammatory reaction to infections by modulating the MAPK signaling pathway.
Zhang [85]	-	Wistar	8	L4 and R4	2 × 10^7^	Salvia miltiorrhizae polysaccharides (SMPs)	SMPs on mastitis induced by *S. aureus* in rats might be related to the inhibition of the activation of the NF-κB and MAPK signaling pathways and thus the inhibition of the expression of inflammatory cytokine genes.

## Data Availability

The data that support the findings of this study are included in this article and available from the corresponding author upon reasonable request.
